# Three-Dimensional-Printed Carbon Nanotube/Polylactic Acid Composite for Efficient Electromagnetic Interference Shielding

**DOI:** 10.3390/polym15143080

**Published:** 2023-07-18

**Authors:** Zhenzhen Xu, Tiantian Dou, Yazhou Wang, Hongmei Zuo, Xinyu Chen, Mingchun Zhang, Lihua Zou

**Affiliations:** 1 School of Textile and Garment, Anhui Polytechnic University, Wuhu 241000, China; xuzhenzhen@ahpu.edu.cn (Z.X.); 15345585302@163.com (T.D.); 18110643193@163.com (Y.W.); 18740356919@163.com (X.C.); 13155600935@163.com (M.Z.); 2 Anhui Province International Cooperation Research Center of Textile Structure Composite Materials, Anhui Polytechnic University, Wuhu 241000, China

**Keywords:** carbon nanotubes, polylactic acid, 3D printing, electromagnetic interference shielding, composites

## Abstract

High-performance electromagnetic interference (EMI) shielding materials with ultralow density and environment-friendly properties are greatly demanded to address electromagnetic radiation pollution. Herein, carbon nanotube/polylactic acid (CNT/PLA) materials with different CNT contents, which exhibit characteristics of light weight, environmental protection and good chemical stability, are fabricated using 3D printing technology, where CNTs are evenly distributed and bind well with PLA. The performances of 3D-printed CNT/PLA composites are improved compared to pure 3D-printed PLA composites, which include mechanical properties, conductive behaviors and electromagnetic interference (EMI) shielding. The EMI shielding effectiveness (SE) of CNT/PLA composites could be improved when the content of CNTs increase. When it reaches 15 wt%, the EMI SE of 3D-printed CNT/PLA composites could get up to 47.1 dB, which shields 99.998% of electromagnetic energy. Meanwhile, the EMI shielding mechanism of 3D-printed CNT/PLA composites is mainly of absorption loss, and it generally accounts for more than 80% of the total shielding loss. These excellent comprehensive performances endow a 3D-printed CNT/PLA composite with great potential for use in industrial and aerospace areas.

## 1. Introduction

Although the widespread use of electronic equipment has brought great convenience to human life, the development of electronic science and technology has made people surrounded by a large amount of electromagnetic radiation, causing great harm to human health [1]. Electromagnetic interference (EMI) shielding could significantly reduce and sometimes even prevent electromagnetic systems and devices from emitting stray EMI signals into the environment. Therefore, the study of efficient electromagnetic shielding materials is imminent and greatly needed to suppress EMI pollution.

Traditional electromagnetic shielding materials are mainly realized by metal and relevant composite materials [2,3]. Although these materials have good electromagnetic shielding performance, there also exist some limitations, such as high density, easy corrosion, and difficulty in adjusting electromagnetic shielding efficiency, which limits their application as multi-functional materials and disables them from meeting the functional requirements of many devices. With the development of plastics, the disadvantages of metal corrosion, heavy weight and difficulty to process are solved. However, plastics are nonconductive and cannot meet the requirements of EMI shielding. In this case, researchers prepared electromagnetic shielding composites by adding conductive fillers [4], such as MXene [5], carbon nanotubes (CNTs) [6,7] and graphene [8] to form conductive networks inside the plastic matrix. Han [7] applied CNTs in reduced GO–CNT vertical edge-rich graphene (rGO–CNT–VG) aerogels and studied its electromagnetic interference shielding behaviors. The results showed that the rGO–CNT–VG/epoxy composites possessed excellent thermal conductivity and an outstanding EMI shielding effectiveness of 56.65 dB, which is 1.9 times higher than that of pure rGO/epoxy composites. Among various conductive fillers, CNTs were broadly used due to their low percolation threshold, perfect electrical conductivity and excellent aspect ratio.

In order to further meet the requirements of lightweight electromagnetic shielding materials, foaming and aerogel preparation technologies were investigated to develop lightweight electromagnetic shielding materials with hole structure [9,10,11]. However, during the preparation process, the unique-sized pores were not easy to control, affecting industrial-scale production. Three-dimensional printing technology, as an additive manufacturing method (AM) of which the structure can be precisely adjusted, has shown significant advantages in the preparation of EMI shielding materials [12,13,14]. In fact, the feedstock to the 3D printer is commonly a kind of thermoplastic polymer filament which is extruded with a PC-controlled moving nozzle, where it is heated above its glass transition temperature. With the 3D printing process, the desired 3D structure, layer by layer, is allowed to form and design. At present, it is known that carbon nanotube polymer composites play an important role in the field of EMI shielding, and the technology for 3D printing carbon nanotube polymer composites is also becoming increasingly used [15,16,17]. However, the dispersion of CNTs not only determines whether the conductive network in the polymer composite can be effectively constructed, but also whether the 3D printing process can proceed smoothly. Therefore, it is necessary to explore the effects of the dispersion of CNTs.

Polylactic acid (PLA) is a green material with good rheological properties, biocompatibility and biodegradability; thus, it has become a widely used raw material of 3D printing [18,19]. Yin et al. [20] prepared PLA/RGO composites using an extrusion process, and a relevant absorber was printed with 3D printer, where the RGO content and geometric parameters of the unit cell were both considered. The results showed that 3D-printed absorber was effective in a broad bandwidth of 4.5–40 GHz to achieve an absorption above 90%. Wu et al. [21] studied the electromagnetic wave absorption capability of CoNC@CF–PLA prepared by 3D printing technology via the introduction of ZIF derivative modified carbon fibers into PLA, and found that the microwave absorption and mechanical properties were both improved. Wang et al. [15] printed PLA scaffolds using the 3D printing method, then dipped CNT on them using the dip coating method, and a hot compress was further used for the 3D CNT/PLA structure. They found that the EMI SE of this kind of structure with 5.0 wt% CNTs could reach 67.0 dB. Huang et al. [22] prepared PLA/PCL/8CNT/IPU composites using the torque rheometer (RM-200C) at 180 °C and 50 rpm for 6 min, and found that the EMI SE increased to 35.6 dB. However, it remains a full challenge to design and prepare lightweight, efficient, easy-to-access and high-strength electromagnetic shielding PLA composites. The combination of CNTs and PLA using 3D printing technology with one step is expected to result in excellent EMI shielding materials.

In this paper, carbon nanotube/polylactic acid (CNT/PLA) composite are prepared using 3D printing technology (fused deposition modeling) to achieve excellent EMI shielding. PLA is used as the substrate, and a conductivity network is provided by adding different contents of CNTs. CNTs are directly deposited in PLA using a high-volatility solvent (i.e., dichloromethane) as dispersion medium to ensure a fast evaporation. The thorough electrical characterization of the 3D-printed samples with an increasing CNT content is performed to evaluate conductivity. In addition, the mechanical behavior of the samples is experimentally investigated and compared by injection and 3D printing technology. Simultaneously, the Fourier transform infrared spectra (FTIR) and EMI shielding properties are also characterized. The purpose of this study is to prepare 3D-printed CNT/PLA composites with a light weight, excellent conductivity and good EMI shielding effects, which is expected to provide a new idea for the development of new EMI shielding materials.

## 2. Materials and Methods

### 2.1. Materials

PLA (6060D) with a density of 1.24 g/cm^3^ and melt flow rate of 5.76 g/10 min (190 °C, 2.16 kg) was purchased from Nature Works (Plymouth, MN, USA); multi-walled CNTs with the diameter of 10–20 nm, length of 10–30 μm, specific surface area of 130 m^2^/g, density of 2.1 g/cm^3^ and electrical conductivity of 10,000 S/m were bought from Beijing Tiannai Co., Ltd. (Beijing, China); and dichloromethane (99.5%) was obtained from Taicang Shanghai Test Reagent Co., Ltd. (Shanghai, China).

### 2.2. The Preparation of CNT/PLA Composites

**Figure 1:**
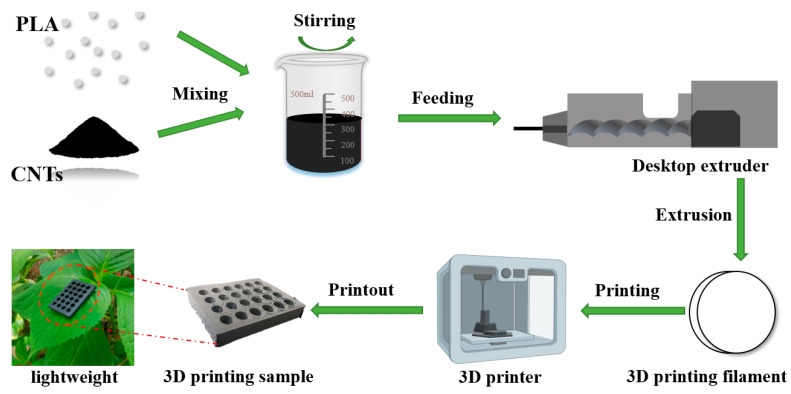
Preparation process flow diagram of CNT/PLA composites.

The preparation process flow diagram of CNTs/PLA composites is given in Figure 1. CNTs and PLA were dried in the oven prior to their use. The mixing process of CNTs and PLA is also a crucial factor which determinates the results of electrical and EMI shielding properties. Herein, to prepare 50 g of 5 wt% CNT/PLA electromagnetic shielding composites, 2.5 g dried CNTs and 47.5 g PLA were weighted, and then dichloromethane was added to mix them evenly using a magnetic mixer. Then, they were dried naturally in the fume hood, cut using the shear machine, and put into a single screw desktop extruder (Type B, Shenzhen Misida Technology Co., Ltd. (Shenzhen, China)) with a feed rate of 2.3 g/min. Finally, a 3D printing filament with a diameter of 1.75 mm was extruded by adjusting the temperature with the range of 170–180 °C and screw speed of 30 rpm. Lastly, the filaments were put into the 3D printer (Sermoon V1, Shenzhen Creative 3D Technology Co., Ltd. (Shenzhen, China)), and then the 5 wt% CNT/PLA samples could be obtained. Similarly, CNT/PLA composites with 10 wt%, 15 wt% and 17 wt% CNT were respectively prepared, according to the above-mentioned methods.

Solidworks software was used to design the 3D model of the EMI shielding composite, and the STL format file was exported. Then, the STL format file was transformed into G-code format file, which facilitated to the layer-by-layer deposition of CNT/PLA composites into the 3D printing component. The 3D printing process parameters were set as follows: nozzle diameter of 0.4 mm, print layer thickness of 0.2 mm, filling density of 20%, printing speed of 30 mm/s, nozzle temperature of 190 °C, hot bed temperature of 55 °C, and the sample dimensions of length × width × thickness: 22.86 mm × 10.86 mm × 3 mm for EMI shielding testing. For the circle hole structure, it was designed with a circle diameter of 4 mm, and the distance between the two adjacent circle holes was 5 mm. A grid was used as the infill pattern, with two layers top/bottom and a 50% filling ratio.

The tensile mechanical performance of the PLA and CNT/PLA samples prepared by injection molding and 3D printing were compared. A micro-injection molding machine (SZS-20, Wuhan Ruiming Experimental Instrument Co., Ltd. (Wuhan, China)) was used to prepare the injected PLA sample with a molding temperature of 190 °C, injection pressure of 100 MPa, holding pressure of 72 MPa and cooling time of 5 s in the mold, which was then taken out and cooled to room temperature.

### 2.3. Characterization

**Figure 2:**
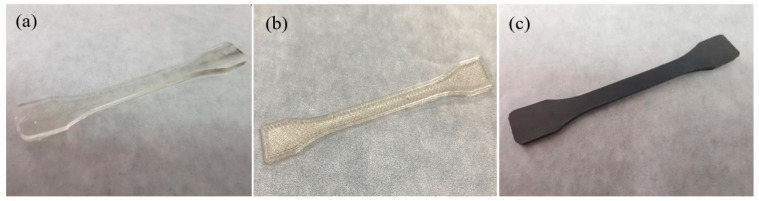
The tensile samples of (**a**) the injected PLA sample; (**b**,**c**) 3D-printed PLA and CNT/PLA samples.

The morphology and microstructure of the samples’ cross-section and longitudinal surface were observed using a scanning electron microscope (SEM, Hitach S-4700, Hitach, Tokyo, Japan), and a thin layer of gold was coated on the surface of the samples before observation. The infrared spectrometer (FTIR, Nicolet NEXUS-670, Thermo Fisher Scientific, Waltham, MA, USA) was used to characterize the types and changes of organic functional groups of CNT/PLA composites (thickness of ~100 μm), with a resolution of 4 cm^–1^ and 32 scans. The tensile strength of the samples was tested using a tensile tester (CSS-88100, Changchun Testing Machine Research Institute, Changchun, China), with the speed of 50 mm/min. Figure 2 displays the tensile samples of PLA and CNT/PLA fabricated by injection molding and 3D printing technology. The effective length scale of the tensile sample was 45 mm, with a width of 5 mm and thickness of 2 mm. It could be seen that the 3D-printed CNT/PLA sample became black compared with that of pure PLA samples which were white and transparent. The average values of the five specimens were obtained. Electrical conductivity (σ, S/m) was calculated through the following equation: σ = L/(R*S), where L is the length (m), R is the resistance (Ω) and S is the cross-sectional area of the sample (m^2^) [23]. R was measured using a digital resistor (Agilent 34401A, Santa Clara, CA, USA) with two probes making contact with the left and right sides. Before the test, the left and right rectangular surfaces (length direction) of were coated with a uniform layer of silver paste to reduce the contact resistance between the probes and 3D-printed samples. For each sample, five measurements were conducted to acquire the averaged values.

The EMI shielding properties of the samples were characterized by the vector network analyzer (P5004A, Keysight, Santa Rosa, CA, USA), with the frequency range of 8.2–12.4 GHz (X-band) using the waveguide method. Before EMI SE testing, the accuracy of the equipment was adjusted using the thru-reflect-line (TRL) calibration technique to guarantee the test results. The S-parameters of transmission loss (S_21_ or S_12_) and forward transmission loss (S_11_ or S_22_) were measured, and the EMI shielding effectiveness due to reflection (SE_R_) and absorption (SE_A_) were further calculated according to the following equations [24]:

*T =* |S_12_|^2^ = *|*S_21_|^2^ (1)

*R* = |S_11_|^2^ = |S_22_|^2^ (2)

*A* = 1 − *R* − *T* (3)

SE_R_ = −10lg(1 − *R*) (4)

SE_A_ = −10lg[(*T*/(1 − *R*)] (5)

*SE_T_* = SE_R_ + SE_A_ (6)

where *T*, *R* and *A* are the transmission, reflection and absorption coefficient, respectively, and *SE_T_* is the total shielding effectiveness.

## 3. Results and Discussion

**Figure 3:**
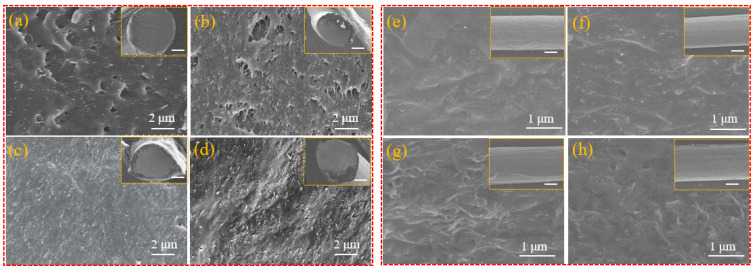
Cross-section and longitudinal morphologies of CNT/PLA samples with different CNT contents, where the sub-images are the thumbnail of the corresponding images, and the bars are 100 μm. (**a**,**e**) 5 wt% CNT/PLA; (**b**,**f**) 10 wt% CNT/PLA; (**c**,**g**) 15 wt% CNT/PLA; (**d**,**h**) 17 wt% CNT/PLA.

Figure 3 shows the cross-section and longitudinal morphologies of the CNT/PLA samples with different contents of CNTs, characterized using SEM. The SEM morphologies in the large images were all obtained from the surface in sub-images. In Figure 3a–d, it could be observed that there are many white strips, which were the electric conductive particles of CNTs, and they were firmly wrapped by PLA. With the increase in CNT content, the distribution density of CNTs in the composite clearly increased, and most of them followed a certain direction and had a good orientation. At the same time, they were uniformly dispersed and there was no obvious agglomeration phenomenon. In Figure 3e–h, the longitudinal images showed that the surface of the CNT/PLA samples was flat and no bubbles were generated. At the same time, with the increase in CNT content, the longitudinal distribution density of CNTs was also increasing, which is conducive to the formation of a conductive network.

**Figure 4:**
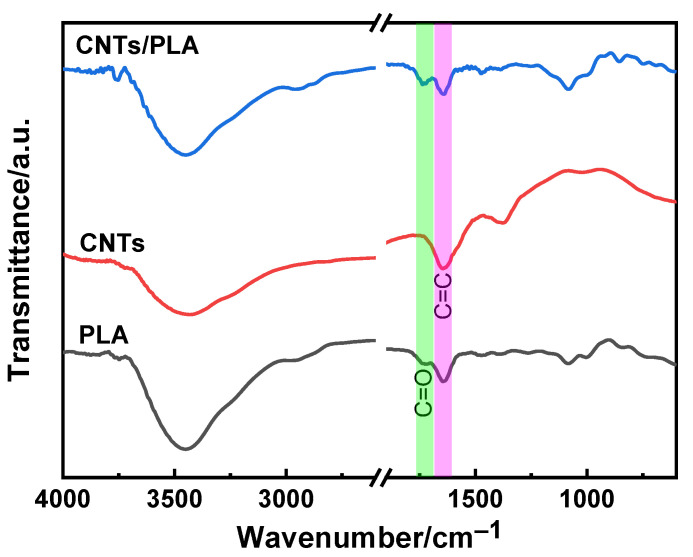
FTIR of PLA, CNTs and 15 wt% CNT/PLA composite.

Figure 4 shows the FTIR images of PLA, CNTs and 15 wt% CNT/PLA composites. In the spectrum of PLA, the characteristic peak at 1720 cm^–1^ is related to a C=O stretching vibration [25]. In the spectrum of CNTs, the characteristic peak at 1645 cm^–1^ is attributed to the C=C stretching vibration [26]. It is obvious that both characteristic peaks of PLA and CNTs were displayed in the CNT/PLA, which demonstrated that PLA and CNTs were well mixed in the composite.

**Figure 5:**
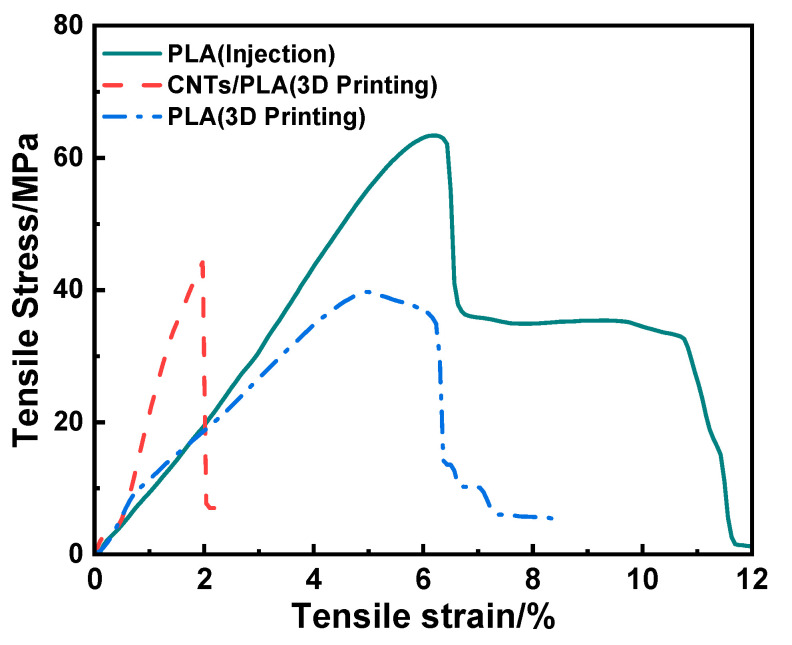
Tensile properties of the injected PLA sample, 3D-printed PLA and 15 wt% CNT/PLA samples.

The tensile properties of the injected PLA sample, 3D-printed PLA and 15 wt% CNTs/PLA samples are respectively provided in Figure 5 and Table 1. It could be seen that the stress of the injected PLA sample reached 63.39 MPa, while that of the 3D-printed PLA sample was only 39.70 MPa. The reason might be that under the action of external force, the molecule chains in the injected PLA sample were more closely connected and oriented, while for the 3D-printed PLA sample, it was stacked layer by layer, which lacked lateral external force. As a result, its tensile stress was lower than that of the injected PLA sample. Moreover, the figure shows that the stress of 3D-printed CNT/PLA composite was higher compared with that of 3D-printed PLA, which was 44.18 MPa, and improved by 11.3%. At the same time, it could be found that the modulus of the CNT/PLA composite was also improved significantly from 630.31 (3D printed PLA) to 1745.45 MPa. Meanwhile, the strain–stress curve showed brittle features compared to the pure PLA composite made using injection and 3D printing methods, with the elongation decreased to only 1.97%. This was because CNTs possess excellent mechanical properties and they are regularly orientated in the PLA matrix, thus CNTs could improve the strength and modulus of the printed composites. Above all, CNTs could reinforce PLA using 3D printing technology, and improve its mechanical properties.

**Figure 6:**
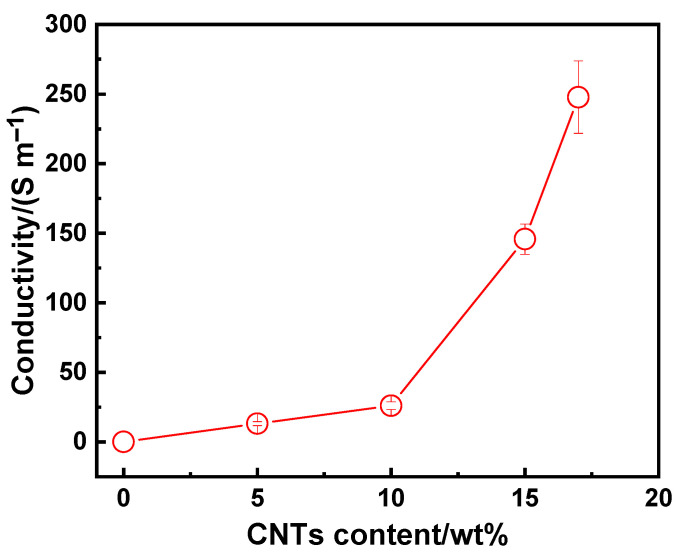
The relationship between the contents of CNTs and electrical conductivity of the CNT/PLA samples.

The electrical conductivity of the composite has an important impact on its shielding performance [27]. Figure 6 shows the relationship between the contents of CNTs and electrical conductivity of the CNT/PLA samples. The variation in the content of CNTs directly affected the electrical conductivity, which resulted from the structure change of the internal conductive network of the composite. For the pure polylactic acid sample, when CNTs were not added, there was no conductivity. With the increase in CNT content, the conductivity gradually increased. When the content was 10 wt%, the conductivity could reach 26.02 S/m, which is higher than that of 60 wt% CNTs with a conductivity of 10 S/m [16]; when it got to 15 wt%, the conductivity increased quickly and the value of 145.74 S/m was obtained, which is comparable to the results of [17], with 82 S/m (graphene at 2 wt% and CNT at 4 wt%). This could be attributed to that the higher content of CNTs, as a better internal conductive network could be efficiently constructed, which could further improve the conductivity of the sample. However, when the CNT content was 17 wt%, although the conductivity improved, it got more difficult to print the CNT/PLA samples due to the nozzle clogging up. Thus, further CNTs were not added to the printed samples.

**Figure 7:**
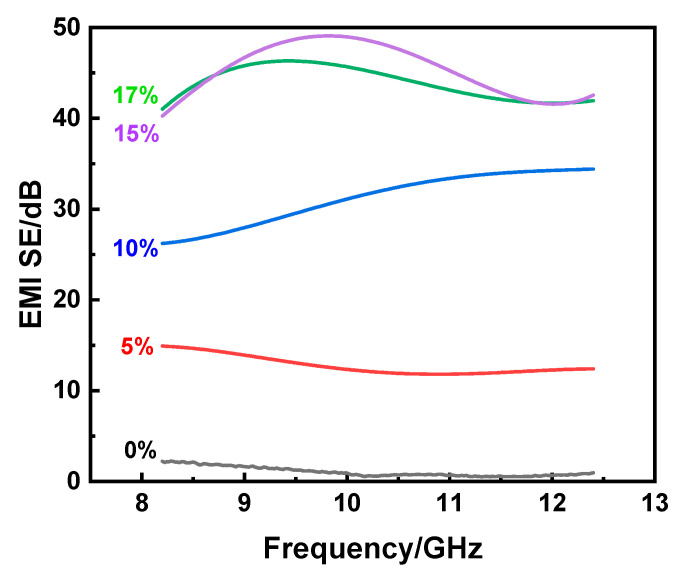
EMI shielding properties of CNT/PLA composites with different CNT contents.

In order to investigate the effect of the CNT content on the EMI shielding performance of CNT/PLA composites, the shielding effectiveness–frequency diagram was drawn. As shown in Figure 7, the EMI SE of the 3D-printed CNT/PLA sample increased with the increase in CNT content. Specifically, when no CNTs were added, the average EMI SE of pure PLA was only 1.05 dB, which was consistent with the study results of [18]. This is mainly because pure PLA is an insulating material and there are not enough carriers to form a connected conductive network, thus it could not effectively shield electromagnetic waves and it is an electromagnetic wave-transparent material. When the dosage of CNTs was 5 wt%, the shielding efficiency reached up to 14.9 dB and 96.76% of electromagnetic energy could be shielded. In addition, when the CNT content was 10 wt%, the EMI SE was 34.42 dB, which is higher than that of 50 wt% CNTs, with an EMI SE of 25.07 dB [16], and could shield 99.96% of electromagnetic energy. When the content of CNTs was 15 wt%, the electromagnetic shielding efficiency was up to 47.1 dB, shielding 99.998% of incident electromagnetic wave, and this was absolutely suitable for commercial applications, which only require 20 dB [17,28]. In the whole frequency range, the shielding efficiency of the prepared 3D-printed samples with the addition of CNTs had exceeded that published in the literature [18], where the EMI SE of 3D-printed MWCNT/GNP (12 wt%) composites was only 13.4 dB. Meanwhile, the density of 3D-printed CNT/PLA composites was only 0.51 g/cm^3^ and could steadily stay on a leaf (Figure 1), which is not only conducive to being lightweight, but could also be used as a highly efficient EMI shielding composite.

**Figure 8:**
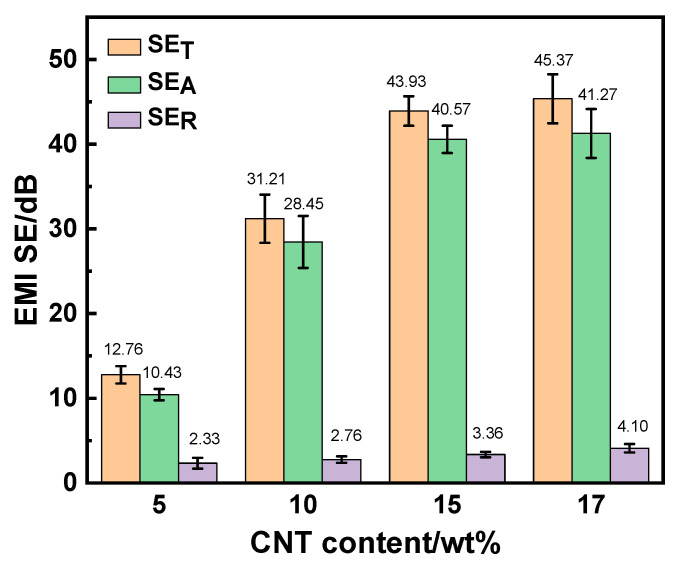
SE_T_, SE_A_ and SE_R_ of CNT/PLA composites with different CNT contents.

In Figure 8, the SE_T_, SE_A_ and SE_R_ of CNT/PLA composites with different CNT contents are shown, where the error bars were also provided. It can be seen that as the content of CNTs increased, both the SE_A_ and SE_R_ increased. Specifically, the SE_A_ increased from 10.43 dB to 28.45 dB, 40.57 dB and 41.27 dB, and the SE_R_ increased from 2.33 dB to 2.76 dB, 3.36 dB and 4.10 dB when the CNT contents increased from 5 wt% to 10 wt%, 15 wt% and 17 wt%, respectively. This meant that the addition of CNTs was much more beneficial to the SE_A_ than the SE_R_. In addition, the EMI shielding mechanism of CNT/PLA composites was mainly absorption loss, generally accounting for more than 80% of the total shielding loss. When the contents of CNTs reached 10 wt%, 15 wt% and 17 wt%, absorption loss exceeded 90%. The higher the proportion of absorption loss was, the stronger the ability of electromagnetic shielding material to absorb electromagnetic waves which incidents into material was. On the other hand, the less the electromagnetic waves were reflected, the less harmful it was to the environment.

## 4. Conclusions

In this study, lightweight 3D-printed CNT/PLA composites with different CNT contents were prepared. CNTs were evenly distributed in CNT/PLA composites and were tightly bound to PLA. The mechanical properties of the 3D-printed CNT/PLA composite was higher than that of the pure 3D-printed PLA composite. Moreover, with the increase in the content of CNTs, the conductive performance was improved, and the conductivity increased. Meanwhile, the electromagnetic shielding effect of CNT/PLA composites was improved with the increase in CNT content. When the content of CNTs reached 15 wt%, the electromagnetic shielding efficiency of 3D-printed CNT/PLA composite was up to 47.1 dB, blocking 99.998% electromagnetic energy. However, when the CNTs were added to make up 17 wt%, the composite was not easily printed any more. The increase in the content of CNTs was conducive to the enhancement of the absorption loss. Additionally, the loss mechanism of 3D-printed CNT/PLA composites was mainly absorption loss, and it generally accounted for more than 80% of the total shielding loss. When the CNT content of CNT reached 10 wt%, 15 wt% and 17 wt%, the proportion of absorption loss was more than 90%. In this case, the prepared 3D-printed CNT/PLA composites had great potential in the field of EMI shielding.

## Figures and Tables

**Table 1:** Tensile properties of the injected PLA, 3D-printed PLA and 3D-printed CNT/PLA.

Sample	Injected PLA	3D-Printed PLA	3D-Printed CNT/PLA
Tensile strength (MPa)	63.39 ± 7.15	39.70 ± 4.79	44.18 ± 5.25
Elastic modulus (MPa)	830.30 ± 9.23	630.31 ± 7.55	1745.45 ± 23.57
Elongation at break (%)	6.23 ± 0.57	5.03 ± 0.49	1.97 ± 0.21

## Data Availability

The data presented in this study are available on request from the corresponding author.

## References

[1] Tian K., Hu D., Wei Q., Fu Q., Deng H. (2023). Recent progress on multifunctional electromagnetic interference shielding polymer composites. J. Mater. Sci. Technol..

[2] Wu B., Zhu H., Yang Y., Huang J., Liu T., Kuang T., Jiang S., Hejna A., Liu K. (2023). Effect of different proportions of CNTs/Fe_3_O_4_ hybrid filler on the morphological, electrical and electromagnetic interference shielding properties of poly(lactic acid) nanocomposites. e-Polymers.

[3] Wanasinghe D., Aslani F. (2019). A review on recent advancement of electromagnetic interference shielding novel metallic materials and processes. Compos. Part B Eng..

[4] Wang Y.-Y., Zhang F., Li N., Shi J.-F., Jia L.-C., Yan D.-X., Li Z.-M. (2023). Carbon-based aerogels and foams for electromagnetic interference shielding: A review. Carbon.

[5] Cheng Z., Chang G., Xue B., Xie L., Zheng Q. (2023). Hierarchical Ni-plated melamine sponge and MXene film synergistically supported phase change materials towards integrated shape stability, thermal management and electromagnetic interference shielding. J. Mater. Sci. Technol..

[6] Cheng H., Pan Y., Wang T., Zhou Y., Qin Y., Liu C., Shen C., Liu X. (2023). Synergistic effects between carbon nanotube and anisotropy-shaped Ni in polyurethane sponge to improve electromagnetic interference shielding. Sci. China Mater..

[7] Han L., Li K., Liu H., Jiao Y., Yin X., Li H., Song Q., Qi L. (2023). Heterogeneous stacking strategy towards carbon aerogel for thermal management and electromagnetic interference shielding. Chem. Eng. J..

[8] Chen Y., Potschke P., Pionteck J., Voit B., Qi H. (2020). Multifunctional Cellulose/rGO/Fe_3_O_4_ Composite Aerogels for Electromagnetic Interference Shielding. ACS Appl. Mater. Interfaces.

[9] Yin H., Bi L., Wu Z., Wang G., Li M., Zhou X., Ji S., Zhang W., Peng Y., Pan J. (2020). 2D foaming of ultrathin MXene sheets with highly conductive silver nanowires for wearable electromagnetic interference shielding applications owing to multiple reflections within created free space. Nano Futures.

[10] Zhao B., Hamidinejad M., Wang S., Bai P., Park C. (2021). Advances in electromagnetic shielding properties of composite foams. J. Mater. Chem. A.

[11] Guo Z., Ren P., Wang J., Hou X., Tang J., Liu Z., Chen Z., Jin Y., Ren F. (2023). Methylene blue adsorption derived thermal insulating N, S-co-doped TiC/carbon hybrid aerogel for high-efficient absorption-dominant electromagnetic interference shielding. Chem. Eng. J..

[12] Aslanzadeh S., Saghlatoon H., Honari M.M., Mirzavand R., Montemagno C., Mousavi P. (2018). Investigation on electrical and mechanical properties of 3D printed nylon 6 for RF/microwave electronics applications. Addit. Manufact..

[13] Lee K.P.M., Baum T., Shanks R., Daver F. (2021). Electromagnetic interference shielding of 3D-printed graphene–polyamide-6 composites with 3D-printed morphology. Add. Manufact..

[14] Wu T., Huan X., Zhang H., Wu L., Sui G., Yang X. (2023). The orientation and inhomogeneous distribution of carbon nanofibers and distinctive internal structure in polymer composites induced by 3D-printing enabling electromagnetic shielding regulation. J. Colloid Interface Sci..

[15] Wang Y., Fan Z.-W., Zhang H., Guo J., Yan D.-X., Wang S., Dai K., Li Z.-M. (2021). 3D-printing of segregated carbon nanotube/polylactic acid composite with enhanced electromagnetic interference shielding and mechanical performance. Mater. Des..

[16] Pei X., Zhao M., Li R., Lu H., Yu R., Xu Z., Li D., Tang Y., Xing W. (2021). Porous network carbon nanotubes/chitosan 3D printed composites based on ball milling for electromagnetic shielding. Compos. Part A Appl. Sci. Manufact..

[17] Shi S., Peng Z., Jing J., Yang L., Chen Y. (2020). 3D Printing of Delicately Controllable Cellular Nanocomposites Based on Polylactic Acid Incorporating Graphene/Carbon Nanotube Hybrids for Efficient Electromagnetic Interference Shielding. ACS Sustain. Chem. Eng..

[18] Spinelli G., Lamberti P., Tucci V., Kotsilkova R., Ivanov E., Menseidov D., Naddeo C., Romano V., Guadagno L., Adami R. (2019). Nanocarbon/Poly(Lactic) Acid for 3D Printing: Effect of Fillers Content on Electromagnetic and Thermal Properties. Materials.

[19] Chizari K., Arjmand M., Liu Z., Sundararaj U., Therriault D. (2017). Three-dimensional printing of highly conductive polymer nanocomposites for EMI shielding applications. Mater. Today Commun..

[20] Yin L., Tian X., Shang Z., Li D. (2019). Ultra-broadband metamaterial absorber with graphene composites fabricated by 3D printing. Mater. Lett..

[21] Wu T., Huan X., Jia X., Sui G., Wu L., Cai Q., Yang X. (2022). 3D printing nanocomposites with enhanced mechanical property and excellent electromagnetic wave absorption capability via the introduction of ZIF-derivative modified carbon fibers. Compos. Part B Eng..

[22] Huang B., Wang Z., Tu J., Liu C., Xu P., Ding Y. (2023). Interfacial distribution and compatibilization of imidazolium functionalized CNTs in poly(lactic acid)/polycaprolactone composites with excellent EMI shielding and mechanical properties. Int. J. Biol. Macromol..

[23] Paleari L., Bragaglia M., Fabbrocino F., Luciano R., Nanni F. (2022). Self-Monitoring Performance of 3D-Printed Poly-Ether-Ether-Ketone Carbon Nanotube Composites. Polymers.

[24] Zou L., Lan C., Zhang S., Zheng X., Xu Z., Li C., Yang L., Ruan F., Tan S.C. (2021). Near-Instantaneously Self-Healing Coating toward Stable and Durable Electromagnetic Interference Shielding. Nanomicro Lett..

[25] Cai Y., Lv J., Feng J. (2012). Spectral Characterization of Four Kinds of Biodegradable Plastics: Poly (Lactic Acid), Poly (Butylenes Adipate-Co-Terephthalate), Poly (Hydroxybutyrate-Co-Hydroxyvalerate) and Poly (Butylenes Succinate) with FTIR and Raman Spectroscopy. J. Polym. Environ..

[26] Varga M., Izak T., Vretenar V., Kozak H., Holovsky J., Artemenko A., Hulman M., Skakalova V., Lee D.S., Kromka A. (2017). Diamond/carbon nanotube composites: Raman, FTIR and XPS spectroscopic studies. Carbon.

[27] He J., Han M., Wen K., Liu C., Zhang W., Liu Y., Su X., Zhang C., Liang C. (2023). Absorption-dominated electromagnetic interference shielding assembled composites based on modular design with infrared camouflage and response switching. Compos. Sci. Technol..

[28] Zou L., Sun Y., Dou T., Yao M., Xu Z., Lan C. (2023). Low-temperature treated polypyrrole coated cotton fabrics for efficient electromagnetic interference shielding. Cellulose.

